# Trends in the study of genetic susceptibility

**DOI:** 10.2478/v10102-010-0024-0

**Published:** 2010-11

**Authors:** Eva Horváthová

**Affiliations:** Cancer Research Institute, Slovak Academy of Sciences, SK-833 91 Bratislava, Slovakia

Genotoxicology investigates the molecular basis of cellular responses to DNA damage. During more than three decades of genetic toxicology testing, a large number of tests with varying sensitivity and specificity have been developed.

These genotoxicity tests can predict:

- the likelihood of a substance to be a (rodent) carcinogen,
- the mechanism of carcinogenic activity of different substances,
- if the results of genotoxicity studies only predict carcinogenicity or they are a distinct hazard endpoint (Nohynek, 2005).

The current genetic toxicity testing batteries represent:

- Ames test which is a component of all genetic testing batteries.
- A mammalian cell mutagenicity assay which should confirm or complete the Ames test.
- Chromosomal aberrations tests which are based on a different endpoint than gene mutations.
- Positive *in vitro* results need to be confirmed by *in vivo* tests; results from test batteries have higher predictive value than results of a single test (Nohynek, 2005).

The human molecular epidemiology represents a new trend in the study of genetic susceptibility and interactions between genetic and environmental factors of risk. Doses and biochemical effects biomonitoring have nowadays a tremendous utility providing an efficient means of measuring human exposure to chemical substances. Human biomonitoring considers all routes of uptake and all sources which are relevant making it an ideal instrument for risk assessment and risk management; blood is by far the most approved matrice. Human biomonitoring can be done for most chemical substances which are in the focus of the worldwide discussion of environmental medicine (Angerer *et al*., 2007). Molecular epidemiology studies of human populations exposed to potential mutagens have the aim to assess the risk of genetic disease or cancer by analysing the relationship between internal exposure and biological effects in target cells under consideration of confounding factors (Faust *et al*., 2004). Human biomonitoring can identify new chemical exposures, trends and changes in exposure, establish distribution of exposure among the general population, identify vulnerable groups and populations with higher exposures and identify environmental risks at specific contaminated sites. The sensitivity of methods moreover enables the elucidation of human metabolism and toxic mechanisms of the pollutants (Angerer *et al*., 2007).

Biomarkers of effect that indicate exposure to a causative agent and that reflect the individual risk of disease are numerous. The most commonly used biomarkers in cancer epidemiology include: measurements of DNA damage, such as DNA breaks, altered bases, bulky adducts; chromosomal aberrations (CA), micronuclei (MN) and sister chromatid exchange (SCE), which are also the results of DNA damage (Kassie *et al*., 2000; Collins, 1998). Chromosomal aberrations and micronuclei are biomarkers of damage due to genetic instability or exposure to environmental mutagens or carcinogens. A recent approach is to associate the biomarkers of genetic susceptibility which take into account cancer susceptibility and interindividual differences in the response to a genotoxic exposure, and the analysis of CA and/or MN, which serves as a biomarker of interactions between the environment and the genetic material of the cell. Information is being gathered on how DNA damage and more particularly the frequency of chromosomal aberrations and/or micronuclei depend on the polymorphisms in genes implicated in xenobiotic metabolism (activation and/or detoxification) and DNA lesion repair. For biomonitoring purposes, numerous confounding factors (age, sex, tobacco consumption, etc.) influence the CA and MN biomarker, and thus associating genetic polymorphisms to CA and MN would be useful to better define the prevention and prediction of risk (Iarmarcovai *et al*., 2007a; Iarmarcovai *et al*., 2007b).

The *in vitro* genetic toxicology tests used for regulatory purposes measure formation of gene mutations and chromosomal changes following DNA damage induced by the compounds under test, and are used to predict the carcinogenic potential of pharmaceuticals, industrial chemicals, food additives and cosmetic ingredients (Kirkland *et al*., 2007). *In vitro* test systems will aid in the identification of the most sensitive species and strains. Also, *in vitro* systems are good models for studying qualitative and quantitative species differences in toxicity. Further, *in vitro* systems are excellent models for characterisation of the mode of action/mechanism for critical effects, but findings need to be validated *in vivo*. *In vitro* systems will aid in the extrapolation from high to low dose and from experimental animals to humans (Holme *et al*., 2002).
